# Synthesis of new pyrrole–pyridine-based ligands using an in situ Suzuki coupling method

**DOI:** 10.3762/bjoc.8.116

**Published:** 2012-07-09

**Authors:** Matthias Böttger, Björn Wiegmann, Steffen Schaumburg, Peter G Jones, Wolfgang Kowalsky, Hans-Hermann Johannes

**Affiliations:** 1 Labor für Elektrooptik am Institut für Hochfrequenztechnik, Technische Universität Braunschweig, Bienroder Weg 94, 38106 Braunschweig, Germany; 2 Institut für Anorganische und Analytische Chemie, Technische Universität Braunschweig, Hagenring 30, 38106 Braunschweig, Germany

**Keywords:** boronic acids, complex chemistry, in situ generation, pyrrole-pyridine, Suzuki coupling

## Abstract

The compounds 6-(pyrrol-2-yl)-2,2‘-bipyridine, 2-(pyrrol-2-yl)-1,10-phenanthroline and 2-(2-(*N*-methylbenz[*d,e*]imidazole)-6-(pyrrol-2-yl)-pyridine were synthesized by using an in situ generated boronic acid for the Suzuki coupling. Crystals of the products could be grown and exhibited interesting structures by X-ray analysis, one of them showing a chain-like network with the adjacent molecules linked to each other via intermolecular N–H^…^N hydrogen bonds.

## Introduction

**Scheme 1:**
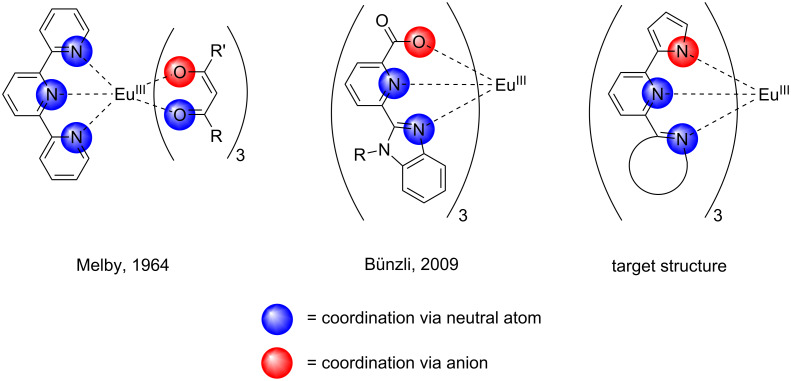
β-diketonate complexes (left), homoleptic complexes (middle) and planned homoleptic complexes of europium(III) (right).

Tridentate ligands have recently received attention in the area of rare-earth-metal complex chemistry [1–5]. Many rare-earth-metal cations, such as europium(III), have the tendency to be nine-coordinating species. Besides their triple positive charge, this coordination sphere must be saturated to achieve high quantum yields when the target is to optimize its luminescent properties [6]. In the “classic” complexes, i.e., the β-diketonates [6–7], this is often achieved by combining three “saturating ligands” that consist of negatively charged β-diketonates with a “neutral ligand”, which is often bidentate or tridentate (Scheme 1, left). The coordination sphere and the positive charge of the rare-earth metal cation are thus saturated and neutralized, respectively. In a recent development, the Bünzli group has succeeded in synthesizing homoleptic complexes, in which only one type of ligand binds to the central europium(III) via two neutral and one negatively charged atom (Scheme 1, middle) [2–4]. As three ligands bind to the rare-earth cation, the resulting complex is neutral, and its coordination number is nine and thereby saturated.

**Scheme 2:**
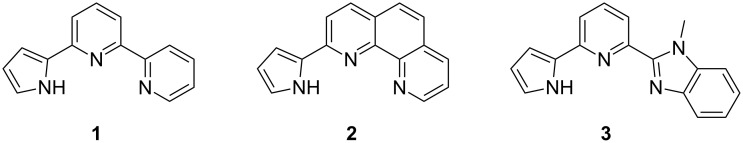
Pyrrole–pyridine-based structures synthesized in this study.

Our interest in this type of complex is driven by its potential analytical application for luminescence degradation measurements on photocatalytic surfaces [8]. Currently, β-diketonate complexes are in use for this analysis, but homoleptic complexes may be advantageous in our opinion, because they only consist of one type of ligand, which is then subjected to photocatalytically produced radicals. Therefore, we tried to broaden the scope by synthesizing a new class of ligands for homoleptic complexes, which should bind to europium(III) via a pyrrolate anion (Scheme 1, right). The decision to choose a negatively charged heterocycle as a binding unit was based on the idea to enlarge the π-system of the ligand, thereby making it possible to absorb longer wavelengths of light (λ > 350 nm). The target structures are shown in Scheme 2.

Structures **1** and **2** comprise substructures of common neutral ligands used in europium complex chemistry [6,9]: 2,2’-bipyridine and 1,10-phenanthroline. Compound **3** comprises a benzimidazole heterocycle, which was also used by the Bünzli group [1–4]. The synthesis of the resulting complexes was to date unsuccessful. We report here on the synthesis of the new structures **1**–**3**.

## Results and Discussion

**Scheme 3:**
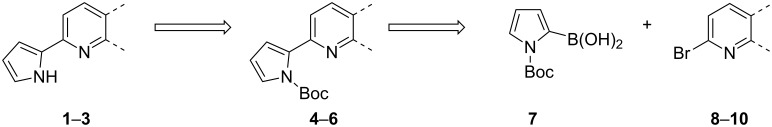
Retrosynthetic approach for structures **1**–**3**.

Our first retrosynthetic approach included a Suzuki coupling of the alpha-substituted boronic acid of Boc-protected pyrrole **7** with the heteroaryl bromides **8**–**10**, as shown in Scheme 3.

**Scheme 4:**
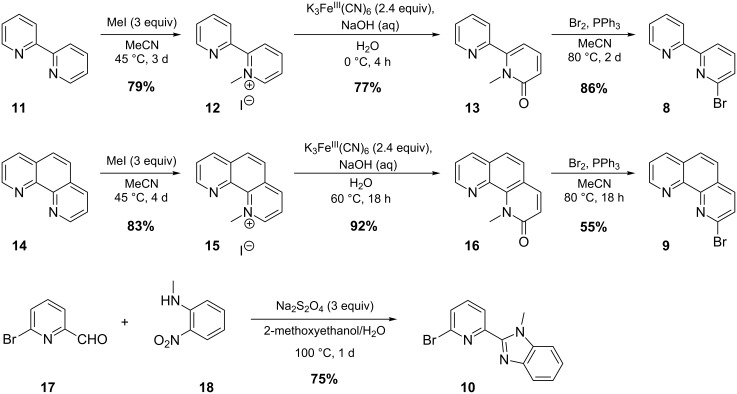
Synthesis of the heteroaryl bromides used in the coupling reaction.

Compound **7** is described in literature [10], but it could not be purified by column chromatography and therefore was not isolated as a pure product. In addition, reports on the stability of this boronic acid show that it is not suitable for long-term storage [10]. We therefore applied a modification of the Suzuki coupling that was also used to prepare [2.2]paracyclophane-derivatives [11]. This comprised the in situ reaction of the freshly prepared boronic acid/ester with the heteroaryl bromides **8**–**10**. These starting compounds could be prepared by using literature procedures, as shown in Scheme 4.

**Scheme 5:**
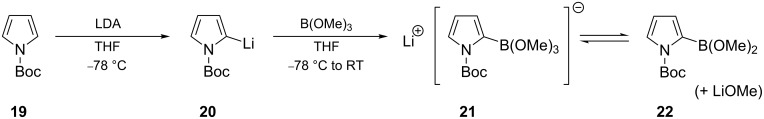
Generation of the borate intermediate **21**/**22**.

Substance **8** was synthesized by following the standard literature procedure [12]. Compound **9** was synthesized by principally the same reaction path [12–13]. 2-Bromopyridine-6-benzimidazole **10** was published in a patent [14], and its preparation was adapted from [4]. The following in situ Suzuki coupling uses Boc-protected pyrrole [15], which was subjected to freshly prepared LDA at −78 °C and quenched with trimethylborate. This gave rise to the intermediate **21**/**22** (Scheme 5).

**Scheme 6:**
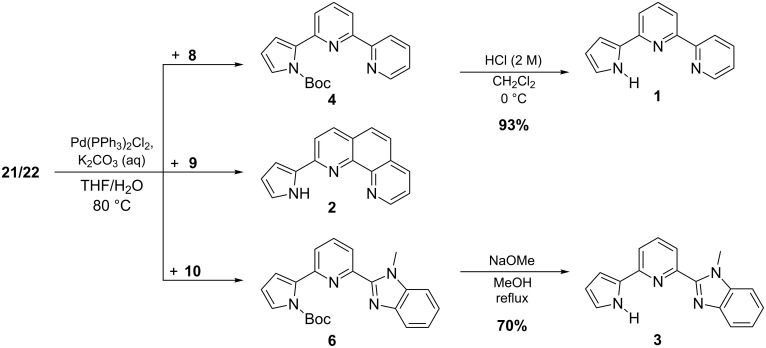
In situ Suzuki coupling reactions of the heteroaryl bromides **8**–**10**.

Afterwards the heteroaryl bromides **8**–**10** were added along with the catalyst and base in aqueous media and the reaction mixture was heated under reflux for several hours. The reaction times were not minimized. In principal the ester can react with the boronic acid, starting with the addition of the aqueous base (K_2_CO_3_). Yet, structure **21** is already activated for the coupling step, since a negative charge is located on the boron atom. In Scheme 6 the coupling reactions are shown; detailed information about the reaction conditions is given in Table 1.

**Table 1:** Reaction conditions used for coupling reactions.

aryl bromide		**19**	LDA	B(OMe)_3_	stirring time^a^	Pd(PPh_3_)Cl_2_	K_2_CO_3_ (1 M)	product	yield
	[equiv]	[equiv]	[equiv]	[equiv]		[mol %]	[equiv]		

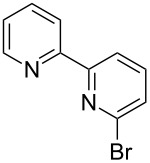 **8**	1.2	1.0	1.1	1.5	15 h	8	1.65	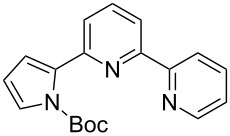 **4**	**84%**
	1.2	1.0	1.1	1.5	15 h	8	1.65		**87%**
	1.0	1.25	1.4	1.9	1.5 h	8	2.0		**89%**

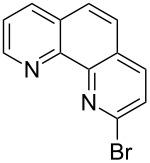 **9**	1.1	1.0	1.1	1.5	1.5 h	8	1.65	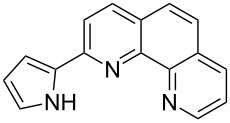 **2**	**41%**
	1.1	1.0	1.1	1.5	16 h	8	1.65		**39%**

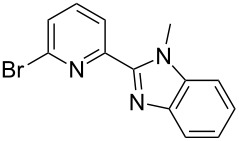 **10**	1.1	1.0	1.1	1.5	0.5 h	5	1.65	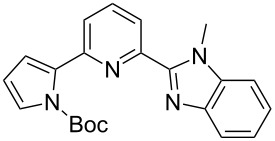 **6**	**52%**
	1.0	1.3	1.25	1.3	0.5 h	5	1.6		**85%**
	1.0	1.5	1.55	1.6	1.2 h	5	2.0		**87%**

^a^Stirring time: After addition of B(OMe)_3_ at 0 °C to RT.

Reaction 1 of aryl bromide **8** to product **4** was repeated under the same conditions and with the same amounts of starting material and catalyst. The workup procedure was changed, which affected the yield only to a very small extent. In the original publication [11] the aryl bromide coupling partner was used in excess. As **19** is by far easier to prepare in large quantities, we altered the protocol and used it in excess, which produced even a slightly better yield than the other way round (Table 1). Finally, deprotection of **4** with hydrochloric acid after the coupling gave **1** in 93% yield. Compound **2** was directly isolated as Boc-deprotected product, but in a much lower yield. We believe the lower yield is caused by the workup procedure, because TLC indicated nearly full consumption of **19**. During column purification with aluminium oxide or silica (both were tried) we noticed smearing of the blue fluorescent product under UV irradiation. Even deactivated aluminium oxide provides enough acidity to split the Boc-moiety, which probably causes the generated amine to partly remain on the column. In the case of **6**, using an excess of **19** lead to better yields, but it has to be mentioned that the workup procedure was also adjusted, thus it cannot be directly compared. Further increasing the equivalents of **19** and the base did not significantly enhance the resulting yield (Table 1). The total reaction time was not systematically minimized. After 20 h under reflux **6** was isolated in 87% yield; a reaction time of 70 h delivered 85% of **6**. Compound **3** was synthesized in 70% yield by deprotection of **6**. In this case another method using sodium methoxide was applied, because reaction with hydrochloric acid did not take place and the unprotected starting material was recovered. We noticed no definite influence on the product yield when using different scales (2 mmol to 12 mmol).

### X-Ray analysis

**Figure 1:**
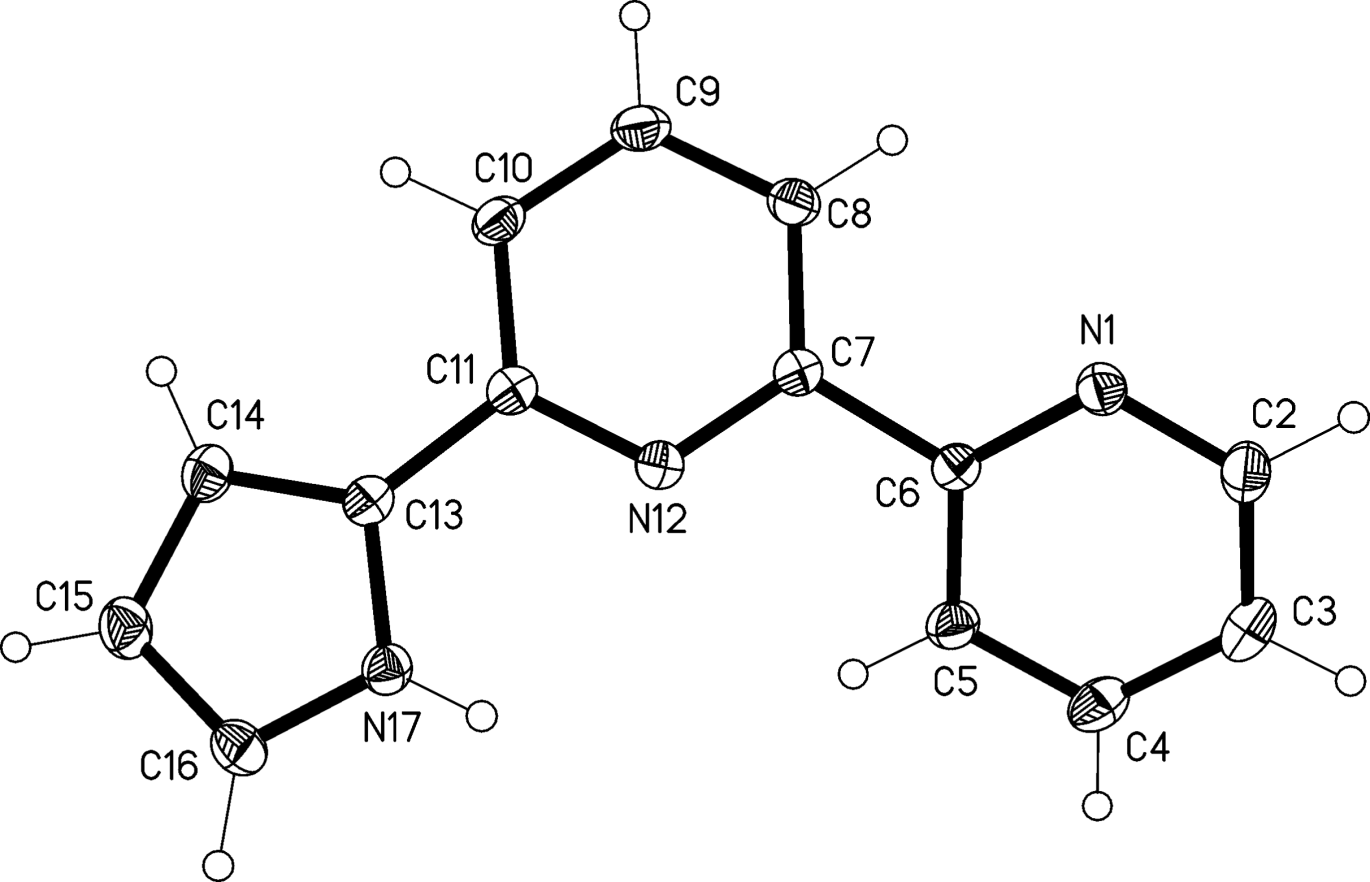
The structure of compound **1** in the crystal. Ellipsoids correspond to 50% probability levels.

**Figure 2:**
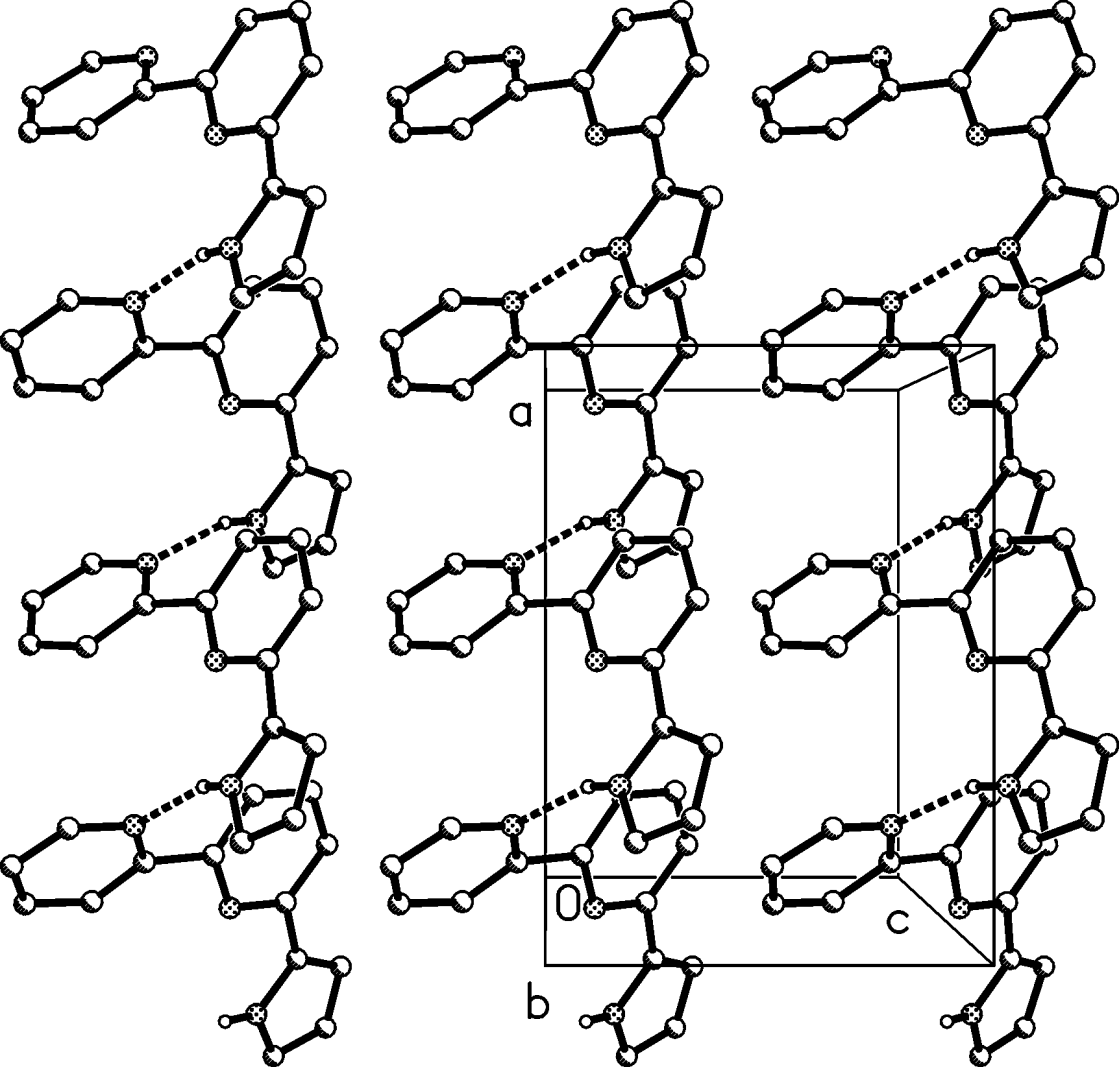
Packing diagram of compound **1**, viewed parallel to the *y*-axis in the range *y* ≈ 1/4. Hydrogen bonds are indicated by dashed lines. Hydrogen atoms not involved in the hydrogen bonds are omitted for clarity. Hydrogen bond details (Å,°) for N17–H17^…^N1: H^…^N 0.88(2), N^…^N 2.861(2), N–H^…^N 158(2), operator 1/2 + *x*, 1 1/2 − *y*, *z*.

The molecular structure of compound **1** is shown in Figure 1. The angles subtended to the central ring are 30° from the pyridyl and 8° from the pyrrolyl substituent. The N^…^N configurations are *trans* for the bipyridyl substructure (N–C–C–N torsion angle −155.8(1)°) but *cis* for the pyrrolylpyridine substructure (torsion angle 3.8(2)°). The former is well-known as a structural preference of 2,2'-bipyridyl systems. A search of the Cambridge Structural Database [16] for 2,2'-pyrrolylpyridines revealed 20 hits, all with *cis* geometry, discounting rigid fused-ring systems and one sterically hindered di-*tert*-butyl system; the corresponding absolute torsion angles ranged from 0 to 25°, mean value 7.5°. The molecular packing of **1** (Figure 2) is determined by a classical hydrogen bond N17–H17^…^N1 involving the peripheral rings, which connects the molecules via the *a*-glide plane to form chains parallel to the *a*-axis.

**Figure 3:**
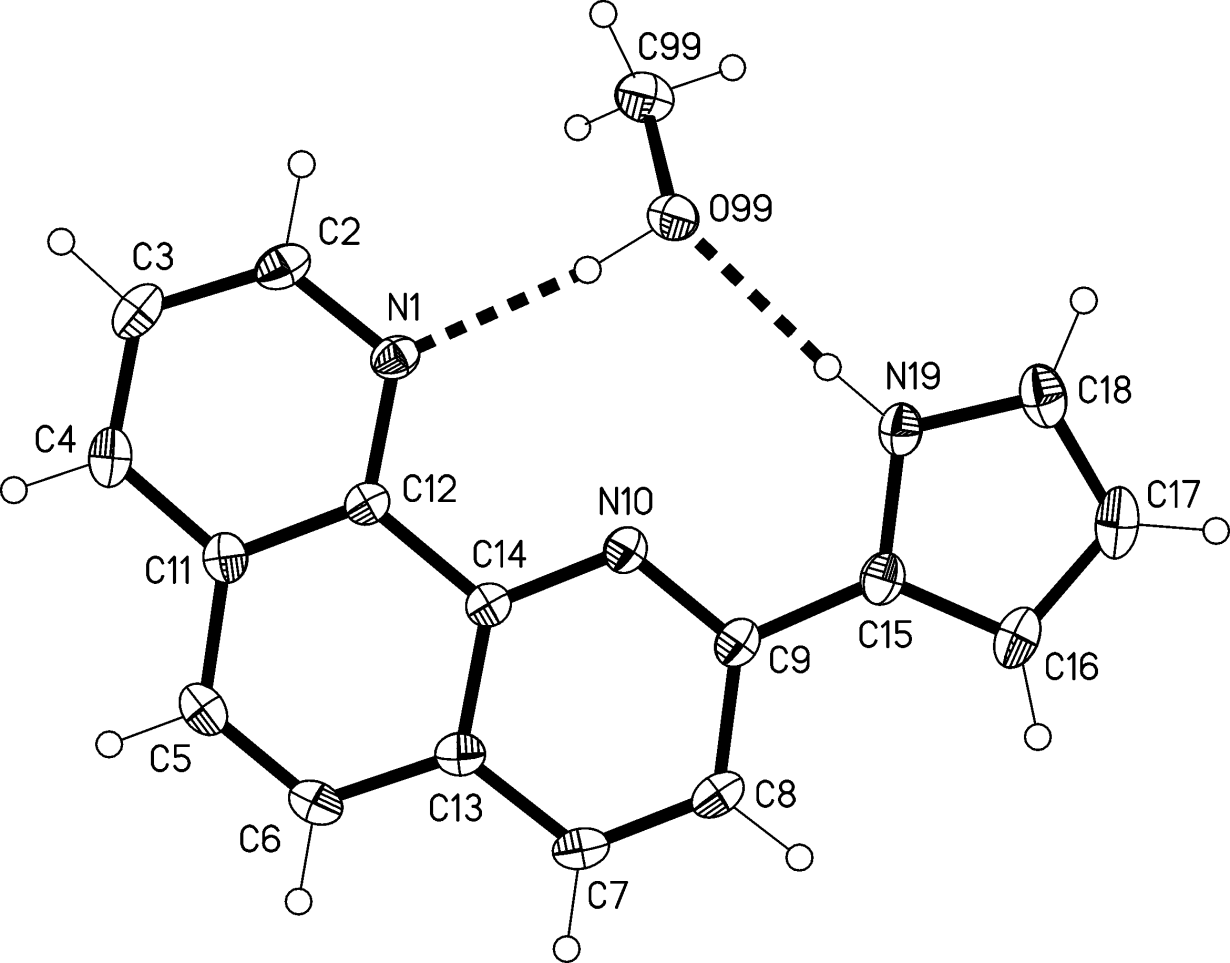
The structure of compound **2**·CH_3_OH in the crystal. Ellipsoids correspond to 50% probability levels. Hydrogen bond details (Å,°) for N19–H19**^…^**O99: H**^…^**O 1.96(2), N**^…^**O 2.839(1), N–H**^…^**O 174(1); for O99–H99**^…^**N1: H**^…^**N 1.93(2), O**^…^**N 2.841(1), N–H**^…^**O 173(2).

**Figure 4:**
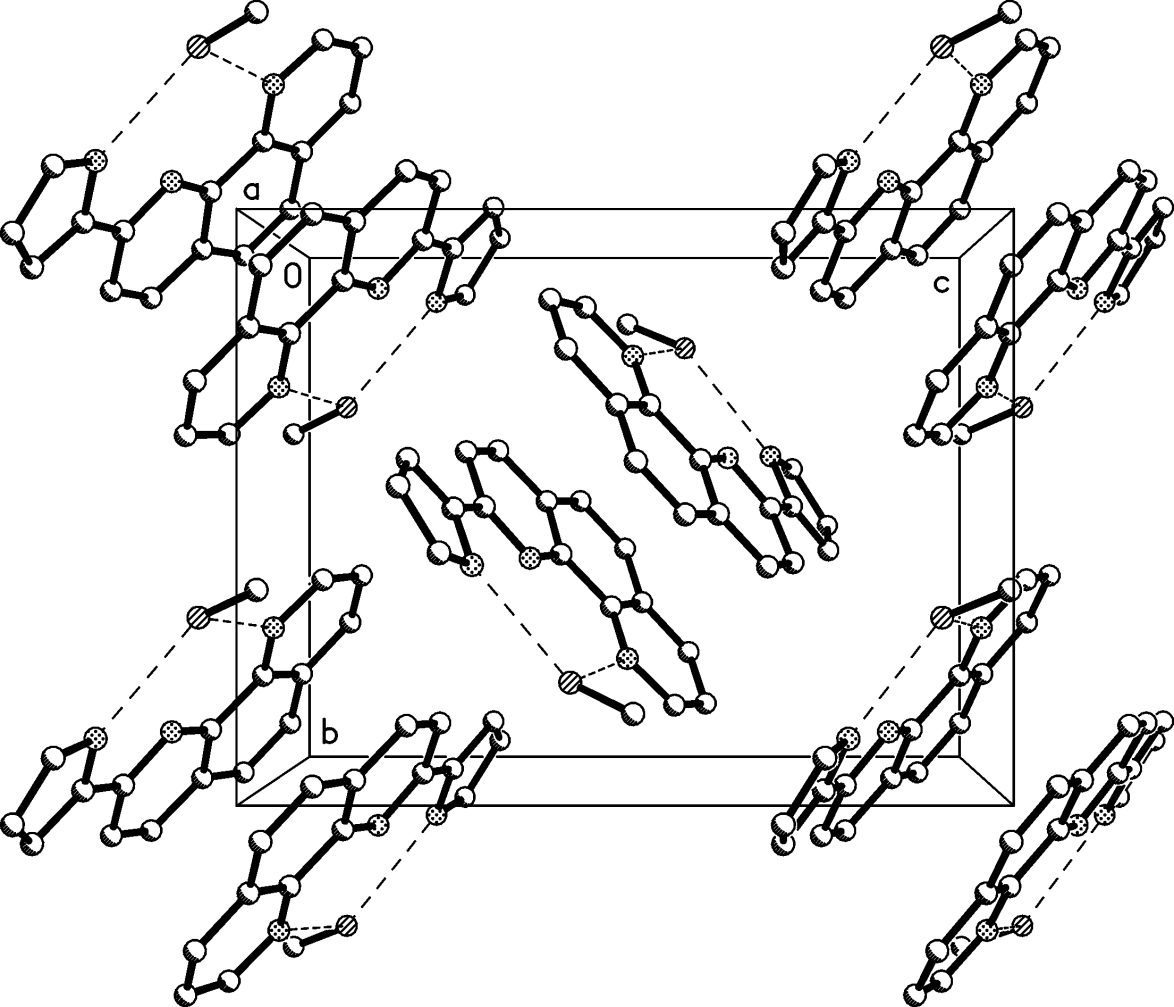
Packing diagram of compound **2**·CH_3_OH showing the formation of inversion-symmetric "stacked" dimers. Hydrogen bonds are shown as thin dashed lines. Hydrogen atoms are omitted for clarity.

The structure of the methanol solvate of compound **2** is shown in Figure 3. The 1,10-phenanthroline ring system is planar (mean deviation 0.01 Å), and the pyrrole ring subtends an interplanar angle of 9° to it. The methanol molecule fits neatly into the “bay” region of the parent molecule, forming classical hydrogen bonds N19–H19**^…^**O99 and O99–H99**^…^**N1. The molecules associate into pairs by ring stacking across inversion centres (Figure 4). The interplanar distance is ca. 3.35 Å.

**Figure 5:**
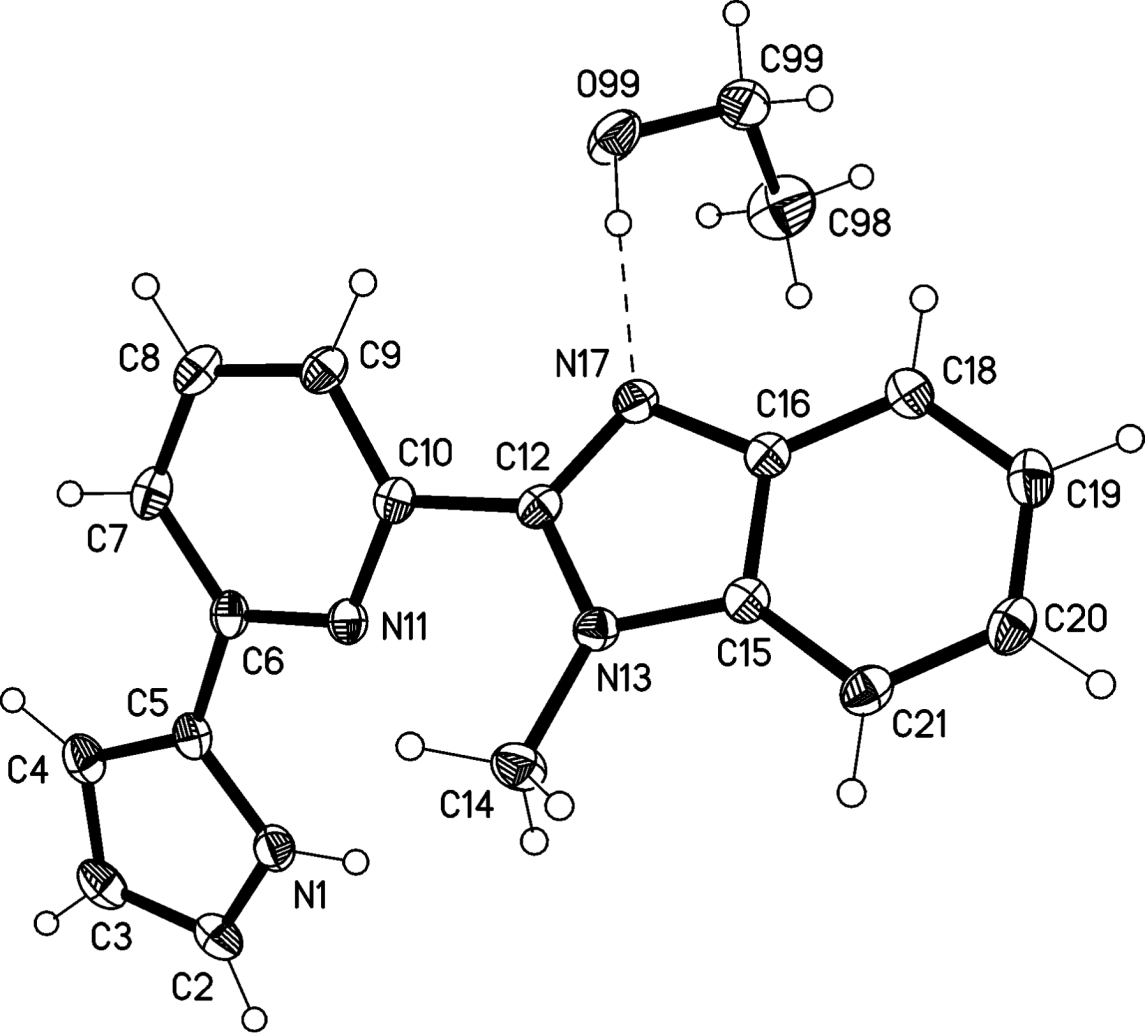
The structure of compound **3**·C_2_H_5_OH in the crystal. Ellipsoids correspond to 50% probability levels. Hydrogen bond details (Å,°) for O99–H99^…^N17: H^…^N 1.91(2), O^…^N 2.813(1), O–H^…^N 174(2).

**Figure 6:**
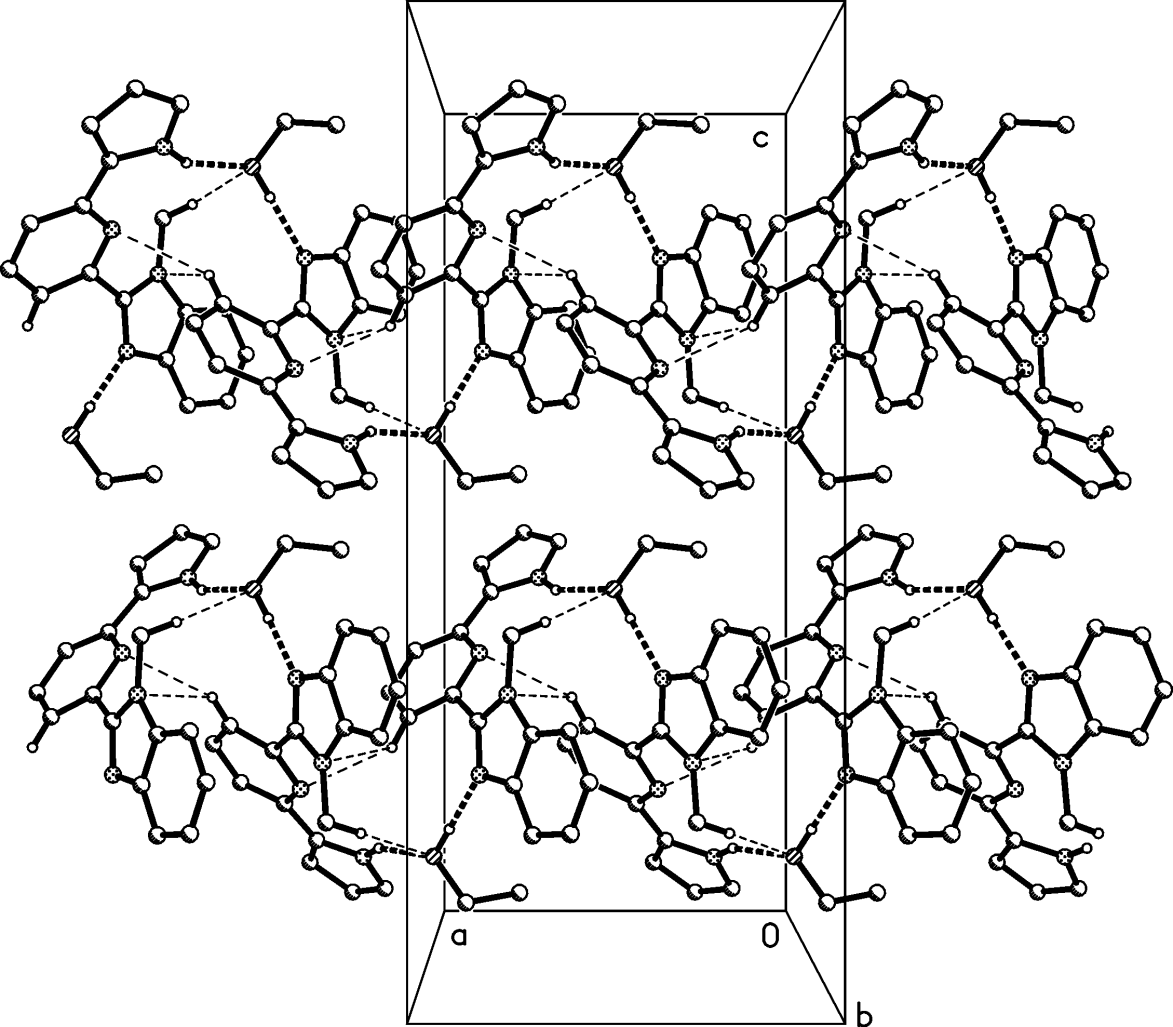
Packing diagram of compound **3**·C_2_H_5_OH. Hydrogen bonds are shown as thick dashed lines. Hydrogen atoms not involved in the hydrogen bonds are omitted for clarity. Hydrogen bond details (Å,°) for N1–H01**^…^**O99: H**^…^**O 2.01(2), N**^…^**O 2.848(1), N–H**^…^**O 154(1), operator −1/2 + *x*, *y*, 1/2 − *z*.

The structure of the ethanol solvate of compound **3** is shown in Figure 5. The central ring is almost coplanar with the pyrrole ring (interplanar angle 9°), but subtends an angle of 38° with the benzimidazole system. Ethanol forms one classical hydrogen bond within the asymmetric unit, acting as a donor, but it also acts as a hydrogen bond acceptor via the *a*-glide plane. The overall effect is to form helical chains of alternating residues of **3** and ethanol, parallel to the *a*-axis (Figure 6).

The crystallographic data of compounds **1** to **3** are summarized in Table 2.

**Table 2:** Crystallographic data for compounds **1**, **2**·CH_3_OH, **3**·C_2_H_5_OH.

compound	**1**	**2**·CH_3_OH	**3**·C_2_H_5_OH

formula	C_14_H_11_N_3_	C_17_H_15_N_3_O	C_19_H_20_N_4_O
*M*_r_	221.26	277.32	320.39
habit	colourless tablet	amber tablet	colourless block
crystallite size (mm)	0.4 × 0.25 × 0.2	0.25 × 0.2 × 0.15	0.4 × 0.3 × 0.15
crystal system	orthorhombic	monoclinic	orthorhombic
space group	*Pna*2_1_	*P*2_1_/*c*	*Pbca*

cell constants:			

*a* [Å]	9.8842(4)	9.7060(3)	9.09323(15)
*b* [Å]	16.1917(6)	10.3442(3)	17.5182(3)
*c* [Å]	7.1501(3)	13.4884(4)	21.2257(4)
α [°]	90	90	90
β [°]	90	91.043(4)	90
γ [°]	90	90	90
*V* (Å^3^)	1144.32	1354.04	3381.19
*Z*	4	4	8
*D*_x_ (g·cm^−3^)	1.284	1.360	1.259
μ (mm^−1^)	0.08	0.09	0.08
*F*(000)	464	584	1360
*T* (°C)	−173	−173	−173
wavelength (Å)	0.71073	0.71073	0.71073
2θ_max_	60	60	60
Refl. measured	34078	50772	125784
Refl. indep.	1791	3936	4949
*R*_int_	0.029	0.028	0.034
parameters	158	100	240
*wR*(*F*^2^, all refl.)	0.084	0.111	0.104
*R*(*F*, >4σ(*F*))	0.031	0.039	0.0406
*S*	1.01	1.03	1.04
max. Δ/ρ (e·Å^−3^)	0.25	0.36	0.36

## Conclusion

The new target structures **1**–**3** were successfully synthesized in good to acceptable yields by applying an in situ variation of the Suzuki coupling as the main reaction step. The crystal structures of these compounds could be obtained by X-ray analysis. They exhibit interesting, but very different structural features: **1** forms chains that consist of molecules directly interconnected by N–H**^…^**N hydrogen bonds, whereas **2** retains the solvent molecule methanol in the “bay” region of the molecule to form stacked dimers. Structure **3** forms a chain-like structure, but retains the solvent molecule ethanol, which connects the molecules via hydrogen bonds. The idea was to synthesize europium(III) complexes containing the new ligands. Since the pyrrole amine is a very weak acid (p*K*_a_ = 23.0) [17] it can only be deprotonated by hard bases, such as *n*-butyllithium or sodium hydride [18]. Therefore we chose to work in water-free conditions with the europium precursors EuCl_3_ (no crystallization water) or Eu[N(SiMe_3_)_2_]_3_, which are commercially available. To date we did not succeed in synthesizing the target complexes depicted on the right-hand side of Scheme 1. This class of compounds may also be of interest for other areas of chemistry. The pyrrole–pyridine structural motif is featured in current studies, owing to its complexation properties towards first-row transition metals (Fe, Co, Ni, Cu, Zn) [18] and ruthenium [19]. The intramolecular proton transfer of these species is also of interest for vibrational spectroscopy measurements [20].

## Experimental

### General

Melting points: Stuart Melting Point SMP3 apparatus, uncorr. Elemental analyses: Vario EL (Elementar Co.). IR: Bruker Tensor 27 spectrometer with a Diamond ATR sampling element. UV–vis: Varian Cary 100 Bio, spectra taken of solutions in spectroscopic grade solvents. NMR: 600 MHz (^1^H), 151 MHz (^13^C): Bruker AV2-600 spectrometer. 200 MHz (^1^H), 50 MHz (^13^C): Varian Mercury Plus 200. ^1^H chemical shifts were recorded with tetramethylsilane (TMS) as the internal standard. ^13^C measurements were taken with the corresponding solvent signal as the reference. *J* values are rounded to 0.1 Hz. Mass spectrometry: Thermofinnigan MAT95XL (EI). TLC: Silica plates (Polygram SIL G/UV 254), aluminium oxide plates (Polygram N/UV 254). Flash chromatography: Silica (Kieselgel 60, Fluka), aluminium oxide (aluminium oxide 90 neutral, Merck). Aluminium oxide, activity III, was made by adding 8% water and shaking the mixture vigorously in a closed flask. All reagents were purchased from Aldrich or Alfa Aesar and used as received. Solvents were purified before use. Dry solvents were purchased from Aldrich or Fluka. Reactions were performed under nitrogen atmosphere unless otherwise stated.

**X-Ray structure determination:** Data collection and reduction: Crystals were mounted in inert oil on glass fibres and transferred to the cold gas stream of the diffractometer (Oxford Diffraction Xcalibur). Measurements were performed with monochromated Mo Kα radiation (λ = 0.71073 Å). No absorption corrections were applied. Structure refinement: The structures were refined anisotropically against *F*^2^ (program SHELXL-97 [21]). *Hydrogen atoms*: OH and NH hydrogens were refined freely; methyl hydrogens as constituents of idealised rigid groups were allowed to rotate but not tip; other H were modelled by using a riding model starting from calculated positions. *Exceptions and special features*: Compound **1**: in the absence of significant anomalous scattering, Friedel opposite reflections were merged and the Flack parameter is thus meaningless. Compound **3**·C_2_H_5_OH: the ethanol molecule is disordered over two positions, but the minor component is occupied only to the extent of 9%. Its OH hydrogen was not located. Similarity restraints for both ethanol orientations were used to improve the stability of refinement. Crystallographic data have been deposited with the Cambridge Crystallographic Data Centre as supplementary publications no. CCDC-871428 (**1**), CCDC-871429 (**2**·CH_3_OH), CCDC-871430 (**3**·C_2_H_5_OH). Copies of the data can be obtained free of charge from http://www.ccdc.cam.ac.uk/data_request/cif.

Synthesis and characterization of the heteroaryl bromides **8** and **9** starting from 2,2’-bipyridine (**11**) and 1,10-phenanthroline (**14**), as well as the synthesis of compound **10** and **19** are shown in Supporting Information File 1. Absorption, excitation and emission spectra of compounds **1**–**3** are included as well.

#### Synthesis of 6-(1-*tert*-butoxycarbonylpyrrol-2-yl)-2,2’-bipyridine (**4**)


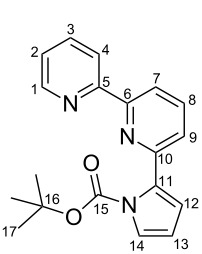


Diisopropylamine (0.36 g, 3.57 mmol, 1.4 equiv) was dissolved in THF (5 mL) and cooled to −80 °C. Whilst the temperature was kept constant, *n*-butyllithium (2.25 mL, 1.6 M in hexane, 3.57 mmol, 1.4 equiv) was added dropwise, and the mixture was stirred for 1 h. Compound **19** (0.55 g, 3.29 mmol, 1.29 equiv) in THF (4 mL) was added dropwise at −80 °C, and the reaction mixture was stirred for another 1 h after which the reaction was quenched with trimethylborate (0.50 g, 4.85 mmol, 1.9 equiv). The mixture was allowed to warm to 0 °C and was stirred for 1.5 h. At room temperature **8** (0.6 g, 2.55 mmol) and Pd(PPh_3_)_2_Cl_2_ (144 mg, 0.2 mmol, 8 mol %) were added. The reaction mixture was heated under reflux and aq K_2_CO_3_ (5.1 mL, 1 M, 5.1 mmol, 2 equiv) was added meanwhile. It was kept at that temperature for 2 h, then cooled to room temperature and diluted with diethyl ether. The organic phase was washed with sat. aq NaCl and the aqueous phase was extracted two times with diethyl ether. The combined organic extracts were then dried over MgSO_4_, filtered and the solvent was removed. The raw product was preadsorbed onto silica. Column chromatography (SiO_2_, hexane/ethyl acetate 3:1, *R*_f_ = 0.4) gave **4** as a yellow resin (0.73 g, 2.27 mmol, 89%). ^1^H NMR (600 MHz, CDCl_3_) δ 8.67 (ddd, ^3^*J*_H,H_*=* 4.8 Hz, ^4^*J*_H,H_ = 1.8 Hz, ^5^*J*_H,H_ = 0.9 Hz, 1H, 1-H), 8.46 (pseudo-dt, ^3^*J*_H,H_* =* 8.0 Hz, *J* = 1.1 Hz, 1H, 4-H), 8.34 (dd, ^3^*J*_H,H_*=* 7.9 Hz, ^4^*J*_H,H_ = 1.0 Hz, 1H, 7-H), 7.81 (dd, ^3^*J*_H,H_ = 7.9 Hz, ^3^*J*_H,H_ = 7.7 Hz, 1H, 8-H), 7.76 (ddd, ^3^*J*_H,H_ = 7.9 Hz, ^3^*J*_H,H_ = 7.6 Hz, ^4^*J*_H,H_ = 1.8 Hz, 1H, 3-H), 7.43 (dd, ^3^*J*_H,H_*=* 7.7 Hz, ^4^*J*_H,H_ = 1.0 Hz, 1H, 9-H), 7.41 (dd, ^3^*J*_H,H_*=* 3.3 Hz, ^4^*J*_H,H_ = 1.8 Hz, 1H, 14-H), 7.28 (ddd, ^3^*J*_H,H_*=* 7.2 Hz, ^3^*J*_H,H_ = 4.6 Hz, ^4^*J*_H,H_ = 1.1 Hz, 1H, 2-H), 6.48 (dd, ^3^*J*_H,H_*=* 3.3 Hz, ^4^*J*_H,H_ = 1.8 Hz, 1H, 12-H), 6.27 (dd, ^3^*J*_H,H_ = 3.3 Hz, ^3^*J*_H,H_ = 3.3 Hz, 1H, 13-H), 1.28 (s, 9H, 17-H) ppm; ^13^C NMR (151 MHz, CDCl_3_) δ 156.0 (s, C-5), 155.0 (s, C-6), 152.1 (s, C-10), 149.4 (s, C-15), 149.0 (d, C-1), 136.7 (d, C-8), 136.7 (d, C-3), 134.3 (s, C-11), 123.6 (d, C-2), 123.6 (d, C-14), 123.1 (d, C-9), 121.3 (d, C-4), 118.8 (d, C-7), 115.6 (d, C-12), 110.5 (d, C-13), 83.5 (s, C-16), 27.4 (q, C-17) ppm; EIMS (70 eV) *m*/*z* (% relative intensity): M^+●^ 321 (5), [M − Boc]^+●^ 222/221/220 (14/100/9); anal. calcd for C_19_H_19_N_3_O_2_: C 71.01, H 5.96, N 13.08; found: C 70.95, H 6.43, N 12.96.

#### Synthesis of 6-(pyrrol-2-yl)-2,2’-bipyridine (**1**)


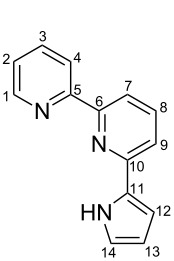


Compound **4** (1.5 g, 4.7 mmol) was dissolved in dichloromethane (110 mL) and cooled to 0 °C. Aqueous hydrochloric acid (27.5 mL, 2 M) was added dropwise under vigorous stirring, upon which the biphasic mixture turned yellow. The organic phase was separated, and the aqueous phase was neutralized with sodium carbonate and then extracted three times with dichloromethane. The combined organic extracts were dried over MgSO_4_ and filtered, and the solution was concentrated. Ethyl acetate (same volume as remaining dichloromethane) was added and the solution was filtered through aluminium oxide. Removal of the solvent yielded **1** as a colorless powder (0.96 g, 4.34 mmol, 93%). Crystals of **1** could be grown by recrystallization from ethanol. Mp 120–121.5 °C; ^1^H NMR (600 MHz, CDCl_3_) δ 8.68 (ddd, ^3^*J*_H,H_ *=* 4.8 Hz, ^4^*J*_H,H_ = 1.8 Hz, ^5^*J*_H,H_ = 0.9 Hz, 1H, 1-H), 8.45 (pseudo-dt, ^3^*J*_H,H_ *=* 8.0 Hz, *J* = 1.1 Hz, 1H, 4-H), 8.15 (dd, ^3^*J*_H,H_*=* 7.7 Hz, ^4^*J*_H,H_ = 1.0 Hz, 1H, 7-H), 7.80 (ddd, ^3^*J*_H,H_ = 7.9 Hz, ^3^*J*_H,H_ = 7.5 Hz, ^4^*J*_H,H_ = 1.8 Hz, 1H, 3-H), 7.75 (dd, ^3^*J*_H,H_*=* 7.9 Hz, ^3^*J*_H,H_ = 7.7 Hz, 1H, 8-H), 7.56 (dd, ^3^*J*_H,H_*=* 7.9 Hz, ^4^*J*_H,H_ = 1.0 Hz, 1H, 9-H), 7.30 (ddd, ^3^*J*_H,H_ *=* 7.5 Hz, ^3^*J*_H,H_ = 4.8 Hz, ^4^*J*_H,H_ = 1.2 Hz, 1H, 2-H), 6.94 (pseudo-dt, *J =* 2.6 Hz, ^4^*J*_H,H_ = 1.4 Hz, 1H, 14-H), 6.76 (ddd, ^3^*J*_H,H_ *=* 3.7 Hz, *J* = 2.5 Hz, ^4^*J*_H,H_ = 1.4 Hz, 1H, 12-H), 6.32 (pseudo-dt, ^3^*J*_H,H_ = 3.6 Hz, *J* = 2.7 Hz, 1H, 13-H), 9.75 (br. s, 1H, N-H) ppm; ^13^C NMR (151 MHz, CDCl_3_) δ 156.2 (s, C-5), 155.0 (s, C-6), 149.9 (s, C-10), 149.1 (d, C-1), 137.4 (d, C-8), 136.7 (d, C-3), 131.6 (s, C-11), 123.6 (d, C-2), 121.0 (d, C-4), 119.7 (d, C-14), 118.2 (d, C-9), 117.9 (d, C-7), 110.3 (d, C-13), 107.3 (d, C-12) ppm; EIMS (70 eV) *m*/*z* (% relative intensity): M^+●^ 222/221/220 (18/100/16); UV–vis (CH_2_Cl) λ_max_, nm (log ε): 307 (4.26), 283 (4.24), 239 (4.23); UV–vis (CH_3_OH) λ_max_, nm (log ε): 309 (4.29), 283 (4.22), 237 (4.23); IR (ATR) 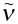: 3126 (m), 3071 (m), 3005 (m), 2970 (m), 2841 (m), 2685 (m), 2551 (m), 1582 (m), 1555 (s), 1455 (s), 1428 (s), 1407 (m), 1328 (w), 1258 (m), 1159 (m), 1126 (s), 1098 (w), 1076 (w), 1061(w), 1033 (m), 1001 (w), 986 (w), 936 (w), 879 (m), 854 (m), 823 (m), 780 (s), 731 (s), 679 (m), 628 (m), 609 (m) cm^−1^; anal. calcd C_14_H_11_N_3_: C 76.00 , H 5.01, N 18.99; found: C 76.20, H 4.75, N 19.18.

#### Synthesis of 2-(pyrrol-2-yl)-1,10-phenanthroline (**2**)


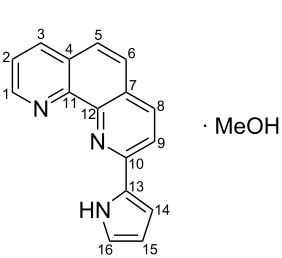


Diisopropylamine (1.11 g, 11.0 mmol, 1.1 equiv) was dissolved in THF (20 mL) and cooled to −80 °C. Whilst the temperature was kept constant, *n*-butyllithium (7.5 mL, 1.6 M in hexane, 12.0 mmol, 1.2 equiv) was added dropwise, and the mixture was stirred for 1 h. Compound **19** (1.67 g, 10.0 mmol) in THF (5 mL) was added dropwise at −80 °C and the reaction mixture was stirred for another 1 h, after which it was quenched with trimethylborate (1.56 g, 15.0 mmol, 1.5 equiv). The mixture was allowed to warm to 0 °C and was stirred for 1.5 h. At room temperature **9** (2.85 g, 11.0 mmol, 1.1 equiv), Pd(PPh_3_)_2_Cl_2_ (555 mg, 0.8 mmol, 8 mol %) and THF (50 mL) were added. The reaction mixture was heated under reflux and aq K_2_CO_3_ (16.5 mL, 1 M, 16.5 mmol, 1.65 equiv) was added meanwhile. It was kept under reflux for 40 h, then cooled to room temperature and diluted with diethyl ether. The organic phase was washed with sat. aq NaCl, and the aqueous phase was extracted two times with diethyl ether. The combined organic extracts were then dried over MgSO_4_ and filtered, and the solvent was removed. The raw product was preadsorbed onto aluminium oxide, activity III. Column chromatography (Al_2_O_3,_ activity III, dichloromethane/ethyl acetate/hexane 1:1:2 → dichloromethane/ethyl acetate 1:1, *R*_f_ (Al_2_O_3_, dichloromethane/ethyl acetate/hexane 1:1:2) = 0.27) gave **2** as a pale yellow solid (1.0 g, 4.1 mmol, 41%). Recrystallization from methanol gave brown crystals of **2**, which retained one solvent molecule as indicated by X-ray and elemental analysis. Mp 119 °C (release of methanol), 137 °C (melting of the remaining solid); ^1^H NMR (600 MHz, CDCl_3_) δ 12.12 (s, 1H, N-H), 8.98 (dd, ^3^*J*_H,H_* =* 4.4 Hz, ^4^*J*_H,H_ = 1.7 Hz, 1H, 1-H), 8.22 (dd, ^3^*J*_H,H_*=* 8.1 Hz, ^4^*J*_H,H_ = 1.6 Hz, 1H, 3-H), 8.10 (d, ^3^*J*_H,H_* =* 8.5 Hz, 1H, 8-H), 7.88 (d, ^3^*J*_H,H_*=* 8.5 Hz, 1H, 9-H), 7.71 (d, ^3^*J*_H,H_*=* 8.7 Hz, 1H, 6-H), 7.64 (d, ^3^*J*_H,H_*=* 8.8 Hz, 1H, 5-H), 7.56 (dd, ^3^*J*_H,H_*=* 8.0 Hz, ^3^*J*_H,H_ = 4.4 Hz, 1H, 2-H), 7.04 (dt, ^3^*J*_H,H_*=* 2.6 Hz, ^4^*J*_H,H_ = 1.4 Hz, 1H, 14-H), 6.90 (ddd, ^3^*J*_H,H_* =* 3.7 Hz, *J* = 2.4 Hz, ^4^J_H,H_ = 1.4 Hz, 1H, 15-H), 6.32 (pseudo-dt, ^3^*J*_H,H_*=* 3.6 Hz, *J* = 2.5 Hz, 1H, 16-H) ppm; ^13^C NMR (151 MHz, CDCl_3_) δ 150.9 (s, C-10), 148.8 (d, C-1), 145.5 (s, C-11), 145.2 (s, C-12), 136.6 (d, C-3), 136.2 (d, C-8), 132.3 (s, C-13), 129.1 (s, C-4), 126.8 (d, C-6), 126.4 (s, C-7), 124.4 (d, C-5), 122.8 (d, C-2), 122.0 (d, C-14), 119.1 (d, C-9), 109.8 (d, C-16), 109.2 (d, C-15) ppm; EIMS (70 eV) *m*/*z* (% relative intensity): [M]^+●^ 247/246/245/244/243 (2/16/100/12/4); UV–vis (CH_2_Cl_2_) λ_max_, nm (log ε_max_): 339 (4.24), 311 (4.31), 235 (3.46); UV–vis (CH_3_OH) λ_max_, nm (log ε_max_): 345 (4.13), 312 (4.24), 237 (4.32) IR (ATR) 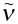:3610 (w), 3185 (m), 3113 (m), 2927 (w), 2820 (w), 1617 (w), 1585 (m), 1554 (m), 1503 (m), 1459 (m), 1423 (m), 1408 (m), 1378 (m), 1338 (w), 1262 (w), 1215 (w), 1145 (m), 1125 (s), 1080 (w), 1030 (s), 936 (w), 882 (w), 839 (s), 779 (s), 728 (s), 679 (s), 626 (m), 606 (s), 569 (m) cm^−1^; anal. calcd. for C_16_H_11_N_3_·CH_3_OH: C 73.63, H 5.45, N 15.15; found: C 73.30, H 5.33, N 15.25.

#### Synthesis of 2-(*N*-methylbenz[*d,e*]imidazo-2-yl)-6-(1-*tert*-butoxycarbonylpyrrol-2-yl)-pyridine (**6**)


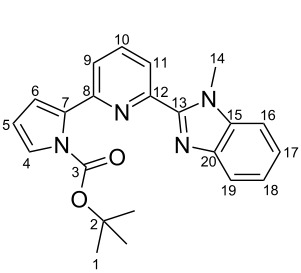


Diisopropylamine (0.22 g, 2.17 mmol, 1.3 equiv) was dissolved in THF (3 mL) and cooled to −80 °C. Whilst the temperature was kept constant, *n*-butyllithium (0.9 mL, 2.5 M in hexane, 2.25 mmol, 1.3 equiv) was added dropwise and the mixture was stirred for 1 h. Compound **19** (0.38 g, 2.27 mmol, 1.3 equiv) in THF (2 mL) was added dropwise at −80 °C and the reaction mixture was stirred for another 1 h, after which it was quenched with trimethylborate (0.23 g, 2.24 mmol, 1.3 equiv). The mixture was allowed to warm to 0 °C and was stirred for 0.5 h. At room temperature **10** (0.49 g, 1.70 mmol), Pd(PPh_3_)_2_Cl_2_ (0.07 g, 0.1 mmol, 6 mol %) and THF (4 mL) were added. The reaction mixture was heated under reflux, and aq K_2_CO_3_ (2.8 mL, 1 M, 2.8 mmol, 1.65 equiv) was added meanwhile. It was kept under reflux for 70 h, then cooled to room temperature and diluted with diethyl ether. The organic phase was washed with sat. aq NaCl and the aqueous phase was extracted two times with diethyl ether. The combined organic extracts were then dried over MgSO_4_ and filtered, and the solvent was removed. Column chromatography (SiO_2_, diethyl ether, *R*_f_ = 0.55) gave **6** as a colorless solid (0.55 g, 1.47 mmol, 85%). mp 125–126 °C; ^1^H NMR (600 MHz, CDCl_3_) δ 8.32 (dd, ^3^*J*_H,H_*=* 7.9 Hz, ^4^*J*_H,H_ = 1.1 Hz, 1H, 11-H), 7.85 (dd, ^3^*J*_H,H_* =* 7.9 Hz, ^3^*J*_H,H_ = 7.8 Hz, 1H, 10-H), 7.83 (ddd, ^3^*J*_H,H_*=* 7.4 Hz, ^4^*J*_H,H_ = 1.6 Hz, ^5^*J*_H,H_ = 0.7 Hz, 1H, 19-H), 7.46 (dd, ^3^*J*_H,H_*=* 7.8 Hz, ^4^*J*_H,H_ = 1.0 Hz, 1H, 9-H), 7.43–7.41 (m, 1H, 16-H), 7.39 (dd, ^3^*J*_H,H_*=* 3.3 Hz, ^4^*J*_H,H_ = 1.8 Hz, 1H, 4-H), 7.33 (ddd, ^3^*J*_H,H_ = 7.5 Hz, ^3^*J*_H,H_ = 7.1 Hz, ^4^J_H,H_ = 1.4 Hz, 1H, 17-H), 7.30 (ddd, ^3^*J*_H,H_ = 7.4 Hz, ^3^*J*_H,H_ = 7.1 Hz, ^4^*J*_H,H_ = 1.3 Hz), 6.50 (dd, ^3^*J*_H,H_ = 3.3 Hz, ^4^*J*_H,H_ = 1.7 Hz, 1H, 6-H), 6.28 (dd, ^3^*J*_H,H _*=* 3.3 Hz, ^3^*J*_H,H_ = 3.3 Hz, 1H, 5-H), 4.25 (s, 3H, 14-H), 1.33 (s, 9H, 1-H) ppm; ^13^C NMR (151 MHz, CDCl_3_) δ 151.6 (s, C-8), 150.1 (s, C-13), 149.7 (s, C-12), 149.3 (s, C-3), 142.5 (s, C-20), 137.2 (s, C-15), 136.8 (d, C-10), 134.0 (s, C-7), 123.9 (d, C-4), 123.2 (d, C-17), 123.1 (d, C-9), 122.6 (d, C-11), 122.5 (d, C-18), 120.0 (d, C-19), 116.0 (d, C-6), 110.6 (d, C-5), 109.9 (d, C-16), 83.9 (s, C-2), 32.8 (q, C-14), 27.5 (q, C-1) ppm; EIMS (70 eV) *m*/*z* (% relative intensity): [M]^+●^ 375/374 (2/6), [M – Boc + methyl]^+●^ 290/289/288/287/286 (10/65/100/67/93), [M − Boc]^+●^ 275/274/273 (6/32/35), [M − Boc-pyrrole + H^+^]^+●^ 209/208/207/206 (3/18/16/12), [*N*-methylbenzimidazole]^+●^ 132/131/130/129 (4/39/2/5); anal. calcd for C_22_ H_22_ N_4_ O_2_: C 70.57, H 5.92, N 14.96; found: C 70.39, H 5.97,N 15.02.

#### Synthesis of 2-(*N*-methylbenz[*d,e*]imidazo-2-yl)-6-(pyrrol-2-yl)-pyridine (**3**)


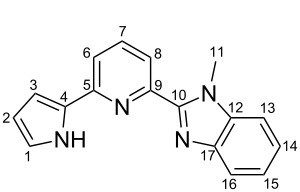


Compound **6** (0.43 g, 1.15 mmol) was dissolved in methanol (35 mL). Sodium methanolate (0.13 g, 2.35 mmol, 2.0 equiv) was added and the solution was stirred for 17 h under reflux. The solvent was removed and the residue was taken up in ethanol (10 mL) and heated again. The mother liquor was then cooled to 0 °C leading to crystallization. Colorless crystals of **3** were collected (0.220 g, 0.802 mmol, 70%). mp 184–187 °C; ^1^H NMR (600 MHz, CDCl_3_) δ 9.83 (s, 1H, N-H), 7.97 (dd, ^3^*J*_H,H_* =* 7.7 Hz, ^4^*J*_H,H_ = 1.0 Hz, 1H, 8-H), 7.85 (m, 1H, 16-H), 7.72 (dd, ^3^*J*_H,H_* =* 8.0 Hz, ^3^*J*_H,H_* =* 7.7 Hz, 1H, 7-H), 7.54 (dd, ^3^*J*_H,H_*=* 8.0 Hz, ^4^*J*_H,H_ = 1.0 Hz, 1H, 6-H), 7.39–7.31 (m, 3H, 13-H, 14-H, 15-H), 7.00 (ddd, ^3^*J*_H,H_*=* 2.6 Hz, *J* = 2.6 Hz, ^4^*J*_H,H_ = 1.4 Hz, 1H, 3-H), 6.77 (ddd, ^3^*J*_H,H_*=* 3.7 Hz, *J* = 2.5 Hz, ^4^*J*_H,H_ = 1.4 Hz, 1H, 1-H), 6.35 (ddd, ^3^*J*_H,H_*=* 3.6 Hz, ^3^*J*_H,H_ = 2.6 Hz, *J* = 2.6 Hz, 1H, 2-H), 4.10 (s, 3H, 11-H) ppm; ^13^C NMR (151 MHz, CDCl_3_) δ 150.8 (s, C-10), 149.7 (s, C-5), 149.3 (s, C-9), 142.5 (s, C-17), 137.3 (d, C-7), 136.9 (s, C-12), 131.4 (s, C-4), 123.2 (d, C-14), 122.6 (d, C-15), 121.7 (d, C-8), 120.2 (d, C-3), 120.0 (d, C-16), 118.2 (d, C-6), 110.5 (d, C-2), 109.9 (C-1), 107.8 (d, C-13), 32.4 (q, C-11) ppm; EIMS (70 eV) *m*/*z* (% relative intensity): [M]^+●^ 276/275/274/273/272 (2/14/80/100/2); UV–vis (CH_2_Cl_2_) λ_max_, nm (log ε_max_): 335 (sh 4.20), 301 (4.41), 230 (4.22); UV–vis (CH_3_OH) λ_max_, nm (log ε_max_): 335 (sh 4.11), 300 (4.41), 232 (4.20); IR (ATR) 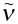: 3170 (m), 3102(m), 2965 (m), 2903 (m), 2858 (m), 1591 (m), 1566 (s), 1473 (s), 1454 (s), 1435 (s), 1391 (m), 1373 (m), 1327 (m), 1250 (m), 1158 (m), 1122 (m), 1083 (m), 1035 (m), 990 (m), 940 (w), 879 (m), 836 (m), 812 (s), 745 (s), 724 (s), 653 (m), 607 (m), 586 (w), 543 (m) cm^−1^; anal. calcd for C_17_H_14_N_4_: C 74.43, H 5.14, N 20.42; found: C 74.31, H 4.86, N 20.33.

## Supporting Information

File 1 Experimental details for known compounds and spectral data for compounds **1**–**3**.

## References

[1] Shavaleev N M, Scopelliti R, Gumy F, Bünzli J-C G (2009). Inorg Chem.

[2] Shavaleev N M, Gumy F, Scopelliti R, Bünzli J-C G (2009). Inorg Chem.

[3] Shavaleev N M, Eliseeva S V, Scopelliti R, Bünzli J-C G (2009). Chem–Eur J.

[4] Shavaleev N M, Eliseeva S V, Scopelliti R, Bünzli J-C G (2010). Inorg Chem.

[5] Bortoluzzi M, Paolucci G, Polizzi S, Bellotto L, Enrichi F, Ciorba S, Richards B S (2011). Inorg Chem Commun.

[6] Melby L R, Rose N J, Abramson E, Caris J C (1964). J Am Chem Soc.

[7] Bauer H, Blanc J, Ross D L (1964). J Am Chem Soc.

[8] Johannes H-H, Kowalsky W, Zoellner M, Böttger M, Wiegmann B (2011). Verfahren und Verwendung lumineszenter Verbindungen zur Messung einer photokatalytischen Oberflächenaktivität.

[9] Filipescu N, Mushrush G W, Hurt C R, McAvoy N (1966). Nature.

[10] Knapp D M, Gillis E P, Burke M D (2009). J Am Chem Soc.

[11] Roche A J, Canturk B (2005). Org Biomol Chem.

[12] Norrby T, Börje A, Zhang L, Åkermark B (1998). Acta Chem Scand.

[13] Halcrow B E, Kermack W O (1946). J Chem Soc.

[14] Nair S K, Planken S P, Plewe M B, Vernier W F, Yang Y, Zhu H (2011). Benzimidazole Derivatives.

[15] Grehn L, Ragnarsson U (1984). Angew Chem, Int Ed Engl.

[16] Allen F H (2002). Acta Crystallogr, Sect B.

[17] Bordwell F G, Drucker G E, Fried H E (1981). J Org Chem.

[18] Ciszek J W, Keane Z K, Cheng L, Stewart M P, Yu L H, Natelson D, Tour J M (2006). J Am Chem Soc.

[19] Nagata T, Tanaka K (2002). Bull Chem Soc Jpn.

[20] Kijak M, Nosenko Y, Singh A, Thummel R P, Brutschy B, Waluk J (2007). J Mol Struct.

[21] Sheldrick G M (2008). Acta Crystallogr, Sect A.

