# Supplementary material for: Synthesis of new pyrrole–pyridine-based ligands using an in situ Suzuki coupling method
Source: Beilstein J Org Chem. 2012 Jul 9;8:1037–47. doi: 10.3762/bjoc.8.116 (PMC3458721; doi:10.3762/bjoc.8.116)
Supplement: File 1 — Experimental details for known compounds and spectral data for compounds 1–3. [file Beilstein_J_Org_Chem-08-1037-s001.pdf]

Supporting Information

for

Synthesis of new pyrrole–pyridine-based ligands using an in situ Suzuki coupling method

Matthias Böttger[1], Björn Wiegmann[1], Steffen Schaumburg[1], Peter G. Jones[2], Wolfgang Kowalsky[1] and Hans-Hermann Johannes[1]*.

Address: [1]Labor für Elektrooptik am Institut für Hochfrequenztechnik, Technische Universität Braunschweig, Bienroder Weg 94, 38106 Braunschweig, Germany and [2]Institut für Anorganische und Analytische Chemie, Technische Universität Braunschweig, Hagenring 30, 38106 Braunschweig, Germany

Email: Hans-Hermann Johannes* - h2.johannes@ihf.tu-bs.de

* Corresponding author

Experimental details for known compounds and spectral data for compounds **1–3**

Experimental details for known compounds

Synthesis of 1-*tert*-butoxycarbonylpyrrole (19)

Compound **19** was synthesized according to the literature [1]. Di-*tert*-butyl dicarbonate (15.8 g, 72.4 mmol) was dissolved in acetonitrile (100 mL). 4-*N,N*-dimethylaminopyridine (1.13 g, 9.25 mmol) and another portion of acetonitrile (50 mL) were added and the solution was stirred for 10 min. Freshly distilled pyrrole (4.07 g, 60.7 mmol) was added and the solution was stirred for 20 h. Subsequently 2-diethylaminoethylamine (2.09 g, 18.0 mmol) was added dropwise, the solution was stirred for 10 min and then added to a mixture of diethyl ether (300 mL) and aq $KHSO_4$ (100 mL, 1 M). The organic phase was washed with aq $KHSO_4$ (50 mL), water (50 mL), aq Na_2CO_3 (100 mL, 1 M) and sat. aq NaCl (100 mL). It was dried over $MgSO_4$ and filtered, and the solvent was removed. Purification by column chromatography (Silica, hexane/diethyl ether 3:1) gave **19** as a colorless oil (9.0 g, 54 mmol, 89%).

^{1}H NMR (200 MHz, $CDCl_3$):

δ [ppm] = 7.25 (t, J = 2.3 Hz, 2H), 6.22 (t, J = 2.3 Hz, 2H), 1.61 (s, 9H).

^{13}C NMR (50 MHz, $CDCl_3$):

δ [ppm] = 149.1, 120.2, 112.1, 83.7, 28.2.

**EA:** $C_9H_{13}NO_2$ (167.21)	calc.	C 64.65	H 7.84	N 8.38
	found	C 64.54	H 8.07	N 8.10.

Synthesis of 1-methyl-2,2'-bipyridinium iodide (12)

Compound **12** was synthesized according to the literature [2]. 2,2'-Bipyridine (5.0 g, 32.0 mmol) was dissolved in acetonitrile (63 mL). Methyliodide (13.6 g, 96.0 mmol, 3.0 equiv) was added, and the solution was then heated to 45 °C and stirred for 3 d. A solid precipitated and was then filtered off, washed with acetonitrile and then dismissed. To the mother liquor was added diethyl ether (25 mL), which led to precipitation of a solid that was filtered off and rinsed once more with diethyl ether. The process was repeated two times after concentration of the mother liquor. The resulting solid was dried in vacuum to give **12** as a light tan powder (7.54 g, 25.3 mmol, 79%).

^{1}H NMR (200 MHz, $CDCl_3$):

δ [ppm] = 9.59 (d, J = 6.2 Hz, 1H), 8.78–8.66 (m, 2H), 8.23 (ddd, J = 7.8 Hz, J = 6.3 Hz, J = 1.5 Hz, 1H), 8.13 (d, J = 8.0 Hz, 1H), 8.08–7.97 (m, 2H), 7.55 (ddd, J = 7.2 Hz, J = 4.8 Hz, 1.6 Hz, 1H), 4.94 (s, 3H).

^{13}C NMR (50 MHz, $CDCl_3$):

δ [ppm] = 152.8, 149.9, 149.5, 147.7, 146.1, 138.2, 130.0, 128.0, 126.7, 125.9, 48.4.

**EA:** $C_{11}H_{11}IN_2$ (298.12)	calc.	C 44.32	H 3.72	N 9.40
	found	C 44.42	H 3.68	N 9.32.

Synthesis of 1-methyl-2,2'-bipyridine-6-one (13)

Compound **13** was synthesized according to the literature [2]. It was not performed under a nitrogen atmosphere. Potassium hexacyanoferrate(III) (13.3 g, 40.3 mmol, 2.4 equiv) was dissolved in water (55 mL) and cooled to 0 °C. Solutions of sodium hydroxide (13.4 g, 335.4 mmol, 20 equiv) in water (50 mL) and **12** (5.01 g, 16.8 mmol) in water (50 mL) cooled to 0 °C were simultaneously added via dropping funnel within 1.5 h. The resulting solution was stirred for another 2.5 h at 0 °C and then warmed to room temperature overnight. Sat. aq NaCl (300 mL) was added and the mixture was extracted several times with dichloromethane (Σ = 500 mL). The combined organic extracts were dried over $MgSO_4$ and filtered, and the solvent was removed. The residue was dissolved in ethyl acetate/methanol (8:2) and filtered through alox. The solvent was removed and the resulting dark brown oil was crystallized overnight. Drying in vacuum gave **13** as dark brown solid (2.41 g, 12.9 mmol, 77%).

^{1}H NMR (200 MHz, $CDCl_3$):

δ [ppm] = 8.69 (ddd, *J* = 4.8 Hz, *J* = 1.8 Hz, *J* = 1.0 Hz, 1H), 7.82 (dt, *J* = 7.7 Hz, *J* = 1.8 Hz, 1H), 7.43 (dt, *J* = 7.8 Hz, *J* = 1.1 Hz, 1H), 7.40–7.3 (m, 2H), 6.62 (dd, *J* = 9.2 Hz, *J* = 1.4 Hz, 1H), 6.18 (dd, *J* = 6.8 Hz, *J* = 1.4 Hz, 1H), 3.42 (s, 3H).

^{13}C NMR (50 MHz, $CDCl_3$):

δ [ppm] = 163.7, 154.1, 149.8, 148.4, 138.6, 137.4, 124.1, 120.4, 108.3, 34.1.

**EA:** $C_{11}H_{10}N_2O$ (186.21)	calc.	C 70.95	H 5.41	N 15.04
	found	C 70.78	H 5.37	N 15.13.

Synthesis of 6-bromo-2,2'-bipyridine (8)

Compound **8** was synthesized according to the literature [2]. Triphenylphosphine (1.97 g, 7.52 mmol, 1.4 equiv) was dissolved in acetonitrile (10 mL). The solution was cooled to 0 °C and bromine (1.12 g, 7.00 mmol, 1.3 equiv) was added dropwise. After stirring for 30 min a yellow precipitate formed. A cooled solution (0 °C) of **13** (1.0 g, 5.37 mmol) in acetonitrile (5 mL) was added quickly. The reaction mixture was heated under reflux for 2 d and then poured onto ice. A white solid formed, which was filtered off and rinsed with dichloromethane. The aqueous phase was neutralized with sat. aq $NaHCO_3$ and extracted three times with dichloromethane (Σ = 250 mL). The combined organic extracts were dried over $MgSO_4$ and filtered, and the solvent was removed. Purification by column chromatography (silica, dichloromethane/ethyl acetate 1:1, R_f (alox) = 0.88) gave **8** as a colorless power (1.08 g, 4.59 mmol, 86%).

^{1}H NMR (200 MHz, $CDCl_3$):

δ [ppm] = 8.68 (ddd, *J* = 4.7 Hz, *J* = 1.6 Hz, *J* = 0.9 Hz, 1H), 8.44–8.35 (m, 2H), 7.82 (dt, *J* = 7.8 Hz, *J* = 1.8 Hz, 1H), 7.67 (t, *J* = 7.8 Hz, 1H), 7.49 (dd, *J* = 7.8 Hz, *J* = 0.7 Hz, 1H), 7.33 (ddd, *J* = 7.5 Hz, *J* = 4.8 Hz, *J* = 1.1 Hz, 1H).

^{13}C NMR (50 MHz, $CDCl_3$):

δ [ppm] = 157.5, 154.7, 149.4, 141.8, 139.4, 137.2, 128.2, 124.5, 121.7, 119.9.

**EA:** $C_{10}H_7BrN_2$ (235.08)	calc.	C 51.09	H 3.00	N 11.92
	found	C 51.35	H 2.80	N 11.87.

Synthesis of 1-methyl-1,10-phenanthrolinium iodide (15)

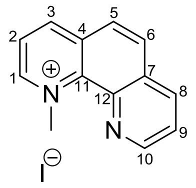

Compound **15** was synthesized according to the literature [2,3]. 1,10-Phenanthroline (5.0 g, 27.7 mmol) was dissolved in acetonitrile (63 mL). Methyliodide (11.8 g, 83.2 mmol, 3.0 equiv) was added and the solution was heated to 45 °C for 4 d. A precipitate formed during the reaction, which was filtered off, rinsed with acetonitrile (50 mL) and then dismissed. To the mother liquor was added diethyl ether (25 mL) upon which a precipitate formed. It was filtered off and rinsed with diethyl ether. The procedure was repeated two times and the collected solids were afterwards recrystallized from ethanol (150 mL). Substance **15** was obtained as yellow solid (7.4 g, 22.9 mmol, 83%).

^{1}H NMR (600 MHz, $(CD_3)_2SO$):

δ [ppm] = 9.60 (dd, $^{3}J_{H,H}$ = 5.9 Hz, $^{4}J_{H,H}$ = 0.7 Hz, 1H, 1-H), 9.41 (dd, $^{3}J_{H,H}$ = 8.2 Hz, $^{4}J_{H,H}$ = 1.3 Hz, 1H, 3-H), 9.32 (dd, $^{3}J_{H,H}$ = 4.3 Hz, $^{4}J_{H,H}$ = 1.8 Hz, 1H, 10-H), 8.80 (dd, $^{3}J_{H,H}$ = 8.2 Hz, $^{4}J_{H,H}$ = 1.8 Hz, 1H, 8-H), 8.44 (d, $^{3}J_{H,H}$ = 8.9 Hz, 1H, 6-H), 8.43 (d, $^{3}J_{H,H}$ = 8.2 Hz, 1H, 2-H), 8.40 (d, $^{3}J_{H,H}$ = 8.8 Hz, 1H, 5-H), 8.06 (dd, $^{3}J_{H,H}$ = 8.2 Hz, $^{3}J_{H,H}$ = 4.2 Hz, 1H, 9-H), 5.28 (s, 3H).

^{13}C NMR (151 MHz, $(CD_3)_2SO$):

δ [ppm] = 151.3 (d, C-1), 149.6 (d, C-10), 146.6 (d, C-3), 140.4 (s, C-12), 137.5 (d, C-8), 137.3 (s, C-11), 132.0 (s, C-4), 131.5 (s, C-7), 130.4 (d, C-6), 126.7 (d, C-5), 125.1 (d, C-9), 124.2 (d, C-2), 54.0 (q, C-13).

**EA:** $C_{13}H_{11}IN_2$ (322.14)	calc.	C 48.47	H 3.44	N 8.70
	found	C 48.67	H 3.36	N 8.41

Synthesis of 1-methyl-1,10-phenanthroline-2-one (16)

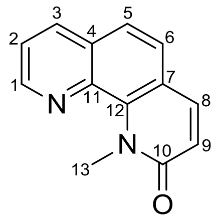

Compound **16** was synthesized according to the literature [2,3]. It was not performed under a nitrogen atmosphere. Potassium hexacyanoferrate(III) (24.6 g, 74.5 mmol, 2.4 equiv) was dissolved in water (100 mL) and heated to 45 °C. A solution of sodium hydroxide (12.4 g, 310 mmol, 20 equiv) in water (100 mL) was added via a dropping funnel and **15** (10.0 g, 31.0 mmol) was added simultaneously in small portions. Water (200 mL) was also added in portions of 50 mL. The reaction mixture was stirred for 18 h at 60 °C upon which a green precipitate formed. After completion the reaction mixture was extracted with dichloromethane (Σ = 700 mL) three times. The combined organic extracts were dried over $MgSO_4$ and filtered, and the solvent was removed. The residue was dissolved in ethyl acetate/methanol (8:2) and filtered through alox. Substance **16** is collected as a brown solid (6.0 g, 28.5 mmol, 92%).

^{1}H NMR (600 MHz, $CDCl_3$):

δ [ppm] = 8.93 (dd, $^3J_{H,H}$ = 4.1 Hz, $^4J_{H,H}$ = 1.9 Hz, 1H, 1-H), 8.16 (dd, $^3J_{H,H}$ = 8.2 Hz, $^4J_{H,H}$ = 1.9 Hz, 1H, 3-H), 7.77 (d, $^3J_{H,H}$ = 9.3 Hz, 1H, 8-H), 7.56 (d, $^3J_{H,H}$ = 8.5 Hz, 1H, 6-H), 7.53 (d, $^3J_{H,H}$ = 8.3 Hz, 1H, 5-H), 7.49 (dd, $^3J_{H,H}$ = 8.2 Hz, $^3J_{H,H}$ = 4.1 Hz, 1H, 2-H), 6.90 (d, $^3J_{H,H}$ = 9.3 Hz, 1H, 9-H).

^{13}C NMR (151 MHz, $CDCl_3$):

δ [ppm] = 164.2 (s, C-10), 147.1 (d, C-1), 140.1 (s, C-11), 139.0 (d, C-8), 137.9 (s, C-12), 136.1 (d, C-3), 130.1 (s, C-4), 126.7 (d, C-6), 122.3 (d, C-5), 122.2 (d, C-9), 121.8 (d, C-2), 120.4 (s, C-7), 37.8 (q, C-13).

**EA:** $C_{13}H_{10}N_2O$ (210.23)	calc.	C 74.27	H 4.79	N 13.33
	found	C 73.97	H 4.69	N 13.22.

Synthesis of 2-bromo-1,10-phenanthroline (9)

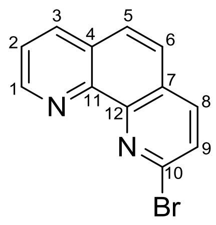

Compound **9** was synthesized according to the literature [2,3]. Triphenylphosphine (5.24 g, 20.0 mmol, 1.4 equiv) was dissolved in acetonitrile (80 mL). The solution was cooled to 0 °C and bromine (2.97 g, 18.6 mmol, 1.3 equiv) was added dropwise over 15 min. After stirring for another 40 min at 0 °C, **16** (3.0 g, 14.3 mmol) dissolved in acetonitrile (50 mL) was added quickly. The reaction mixture was heated under reflux for 18 h and then poured onto ice. It was filtered and the aqueous phase was neutralized with sat. aq $NaHCO_3$ and extracted two times with dichloromethane (Σ = 1.0 L). The combined organic extracts were dried over $MgSO_4$ and filtered, and the solvent was removed. Purification by column chromatography (alox, dichloromethane/ethyl acetate 10:1, R_f = 0.68) gave **9** as a colorless solid (2.04 g, 7.87 mmol, 55%).

^{1}H NMR (600 MHz, $CDCl_3$):

δ [ppm] = 9.23 (dd, $^{3}J_{H,H}$ = 4.3 Hz, $^{4}J_{H,H}$ = 1.8 Hz, 1H, 1-H), 8.25 (dd, $^{3}J_{H,H}$ = 8.1 Hz, $^{4}J_{H,H}$ = 1.7 Hz, 1H, 3-H), 8.06 (d, $^{3}J_{H,H}$ = 8.3 Hz, 1H, 8-H), 7.82 (d, $^{3}J_{H,H}$ = 8.8 Hz, 1H, 5-H), 7.76 (d, $^{3}J_{H,H}$ = 8.3 Hz, 1H, 9-H), 7.76 (d, $^{3}J_{H,H}$ = 8.7 Hz, 1H, 6-H), 7.65 (dd, $^{3}J_{H,H}$ = 8.1 Hz, $^{3}J_{H,H}$ = 4.3 Hz, 1H, 2-H).

^{13}C NMR (151 MHz, $CDCl_3$):

δ [ppm] = 150.8 (d, C-1), 146.7 (s, C-12), 145.0 (s, C-11), 142.5 (s, C-10), 138.1 (d, C-8), 136.0 (d, C-3), 128.9 (s, C-4), 127.9 (d, C-9), 127.4 (s, C-7), 127.1 (d, C-5), 125.8 (d, C-6), 123.4 (d, C-2).

**EA:** $C_{12}H_7BrN_2$ (259.10)	calc.	C 55.63	H 2.72	N 10.81
	found	C 56.18	H 2.50	N 10.71.

Synthesis of 2-bromo-6-(*N*-methyl-benz[*d,e*]imidazo-2-yl)-pyridine (10)

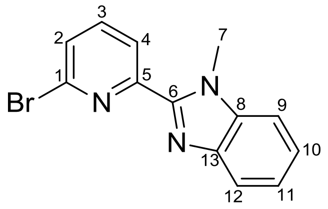

Compound **10** was synthesized according to a literature procedure for a similar derivative [4]. *N*-methyl-nitroaniline (4.89 g, 31.5 mmol) and 6-bromopyridine-2-carbaldehyde (5.86 g, 31.5 mmol) were dissolved in a mixture of 2-methoxyethanol (140 mL) and water (35 mL) and the solution was saturated with nitrogen. Sodium dithionite (85%, 19.4 g, 94.8 mmol, 3.0 equiv) was added and the mixture was stirred for 1 d under reflux. After cooling, the formed precipitate was filtered off and the solvent of the mother liquor was removed. The resulting solid was taken up in a

biphasic mixture of ethyl acetate/water (1:1). The aqueous phase was extracted with ethyl acetate (Σ = 700 mL) several times. The combined organic extracts were then dried over $MgSO_4$ and filtered, and the solvent removed. The residue was dissolved in dichloromethane and filtered through alox (III) (R_f = 0.46). Removal of the solvent gave **10** as yellow crystalline solid (6.8 g, 23.6 mmol, 75%).

mp: 148–149 °C

^{1}H NMR (600 MHz, $CDCl_3$):

δ [ppm] = 8.38 (dd, $^3J_{H,H}$ = 7.8 Hz, $^4J_{H,H}$ = 0.9 Hz, 1H, 4-H), 7.82 (ddd, $^3J_{H,H}$ = 7.9 Hz, $^4J_{H,H}$ = 1.2 Hz, $^5J_{H,H}$ = 0.8 Hz, 1H, 12-H), 7.69 (dd, $^3J_{H,H}$ = 7.9 Hz, $^3J_{H,H}$ = 7.8 Hz, 1H, 3-H), 7.52 (dd, $^3J_{H,H}$ = 7.9 Hz, $^4J_{H,H}$ = 0.9 Hz, 1H, 2-H), 7.44 (ddd, $^3J_{H,H}$ = 8.2 Hz, $^4J_{H,H}$ = 1.3 Hz, $^5J_{H,H}$ = 0.7 Hz, 1H, 9-H), 7.36 (ddd, $^3J_{H,H}$ = 8.0 Hz, $^3J_{H,H}$ = 7.1 Hz, $^4J_{H,H}$ = 1.2 Hz, 1H, 10-H), 7.32 (ddd, 1H, $^3J_{H,H}$ = 7.9 Hz, $^3J_{H,H}$ = 7.1 Hz, $^4J_{H,H}$ = 1.3 Hz, 1H, 11-H), 4.27 (s, 3H, 7-H).

^{13}C NMR (151 MHz, $CDCl_3$):

δ [ppm] = 151.1 (s, C-1), 148.4 (s, C-6), 142.4 (s, C-13), 140.5 (s, C-5), 139.1 (d, C-3), 137.3 (s, C-8), 128.0 (d, C-2), 123.7 (d, C-10), 123.2 (d, C-4), 122.9 (d, C-11), 120.1 (d, C-12), 110.0 (d, C-9), 32.7 (q, C-7).

MS (EI, 70 eV):

m/z (%): 290/289/288/287/286 (10/64/100/67/93) $[M]^{+\bullet}$.

**EA:** $C_{13}H_{10}BrN_3$ (288.14)	calc.	C 54.19	H 3.50	N 14.58
	found	C 54.33	H 3.43	N 14.65.

Spectral data for compounds 1–3

The absorption spectra of compounds **1** to **3** measured in dichloromethane and methanol are shown in Figure S1 to Figure S3. The excitation and emission spectra are depicted in Figure S4 to Figure S6.

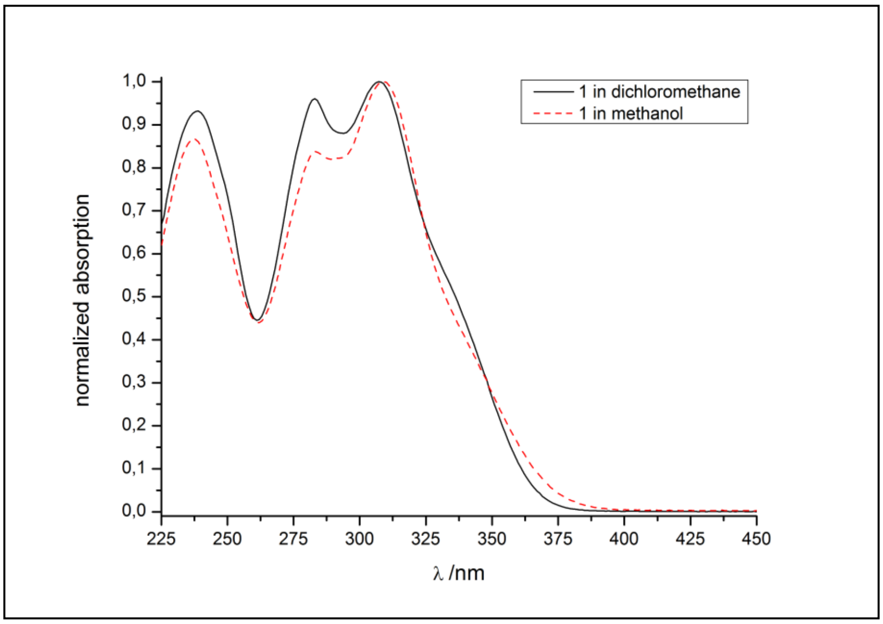

Figure S1: Absorption spectra of **1** in dichloromethane and methanol.

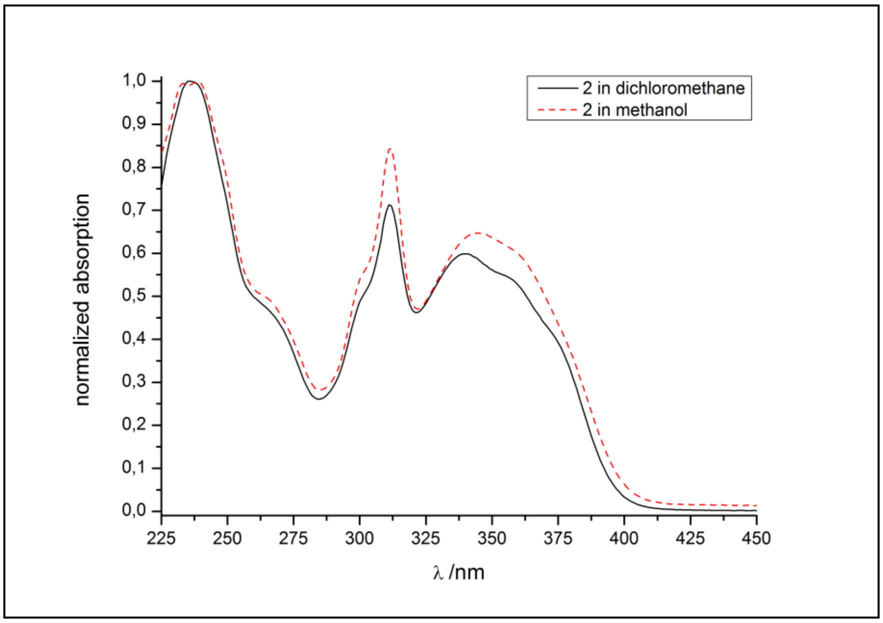

Figure S2: Absorption spectra of **2** in dichloromethane and methanol.

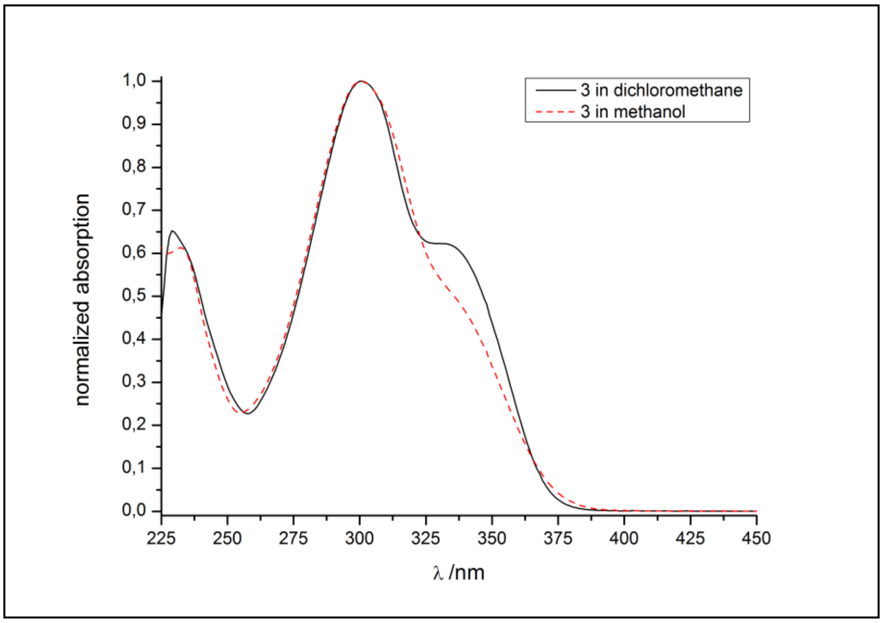

Figure S3: Absorption spectra of **3** in dichloromethane and methanol.

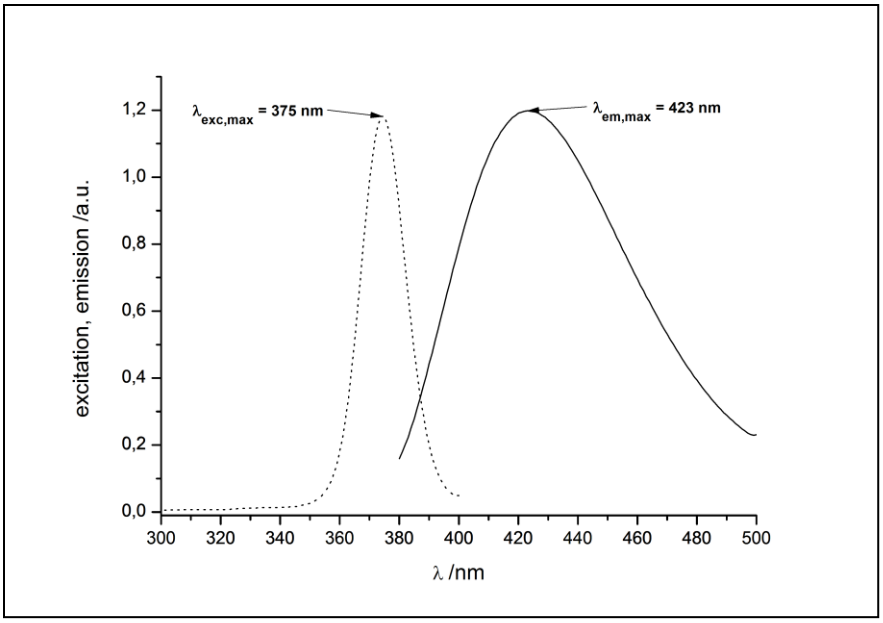

Figure S4: Excitation (dotted line) and emission (solid line) spectra of compound **1**.

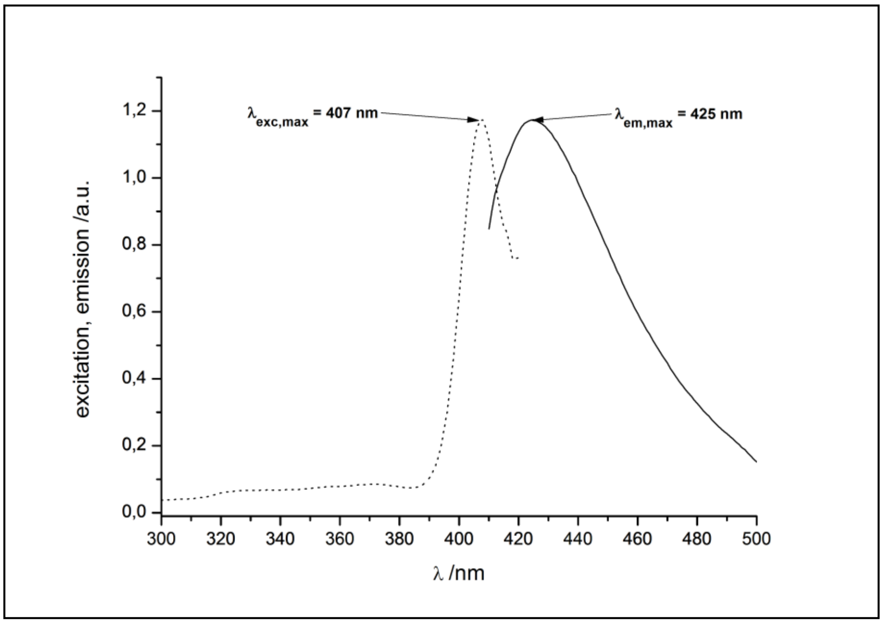

Figure S5: Excitation (dotted line) and emission (solid line) spectra of compound **2**.

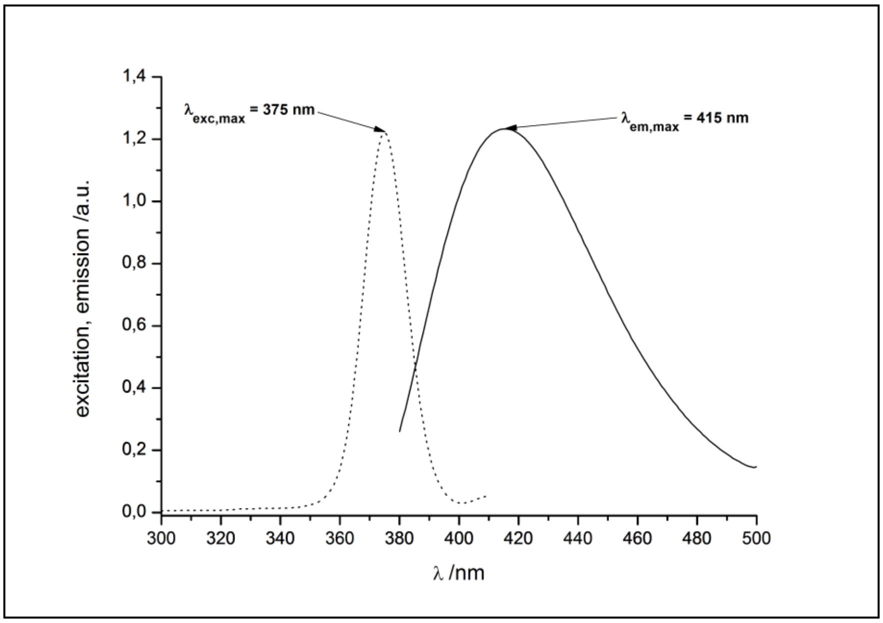

Figure S6: Excitation (dotted line) and emission (solid line) spectra of compound **3**.

References

[1] Grehn, L.; Ragnarsson, U. *Angew. Chem. Int. Ed. Engl.* **1984,** *23,* 296–301. doi:10.1002/anie.198402961

[2] Norrby, T.; Börje, A.; Zhang, L.; Åkermark, B. *Acta Chem. Scand.* **1998,** *52,* 77–85. doi:10.3891/acta.chem.scand.52-0077

[3] Halcrow, B. E., Kermack, W. O. *J. Chem. Soc.* **1946,** 155–157. doi:10.1039/JR9460000155

[4] Shavaleev, N. M.; Eliseeva, S. V.; Scopelliti, R.; Bünzli, J.-C. G. *Inorg. Chem.* **2010,** *49,* 3927–3936. doi:10.1021/ic100129r
